# Informed Self-Placement: A Case Study of the Florida College System

**DOI:** 10.1007/s10755-024-09777-7

**Published:** 2025-01-28

**Authors:** Amanda N. Nix, Shouping Hu

**Affiliations:** 1 Collaborative Lab for the Advancement of Student Success, Florida State University, Tallahassee, FL USA; 2 Anne Spencer Daves College of Education, Health, and Human Sciences, Florida State University, Tallahassee, FL USA

**Keywords:** Advising, Informed self-placement, Directed self-placement, Multiple measures, Community colleges

## Abstract

Traditional college advising, whereby advisors provide course assignments according to standardized placement test scores, has undergone major transformation in recent years. New placement models, like “informed self-placement” based on multiple measures of documented student achievement, are growing in popularity but remain understudied. To address this gap in scholarship, we took advantage of a unique opportunity presented by statewide reform in Florida to explore how Florida College System advisors described the implementation of informed self-placement and multiple measures between 2014 and 2019, paying special attention to emergent challenges and corresponding solutions. Our findings, drawn from the perspectives of 275 advisors representing 19 different institutions shared during semi-structured focus group sessions, revealed that advisors used a combination of high school experiences, non-cognitive factors, and continued placement testing to inform course recommendations that students could then choose to act upon or ignore when registering for their classes. We also observed several challenges (i.e., increased workload, insufficient information, and student resistance) that advisors managed through group advising, advanced preparation, and self-diagnostic tools.

Standardized placement tests have traditionally played a key role in community college advising, particularly when it comes to identifying students in need of remediation. Less than 15 years ago, most public, two-year colleges relied exclusively on math and English placement test scores to assign students to the appropriate level of coursework (Rutschow & Mayer, 2018). However, research now demonstrates that placement tests commonly misplace students, often into courses that are less rigorous than they can manage (Belfield & Crosta, 2012; Scott-Clayton, 2012; Scott-Clayton et al., 2014).

To improve placement accuracy, reformers have begun to experiment with different models (Hodara et al., 2012). One alternative has been to use multiple measures of documented student achievement to inform the advising and placement process. Under this model, college advisors supplement placement test scores with a variety of other measures—like a student’s high school grade point average (GPA) and the highest level of mathematics completed in high school—when generating their course recommendations. The value of these multiple measures is that they provide advisors with a more complete picture of their students’ academic preparation so that “rusty skills or a bad day do not relegate students to remedial coursework they may not need” (Burdman, 2012, p. 17). Multiple measures placement has grown quickly in popularity, with more than half of colleges now using additional information to inform the advising process (Rutschow & Mayer, 2018). Over the past several years, limited access to in-person, proctored placement tests due to the COVID-19 pandemic has only further accelerated the widespread adoption of multiple measures placement (Bickerstaff et al., 2021).

As scholars observe these trends, many have responded with quantitative research projects to determine which multiple measures are the most useful and whether multiple measures positively impact student outcomes, especially in community college settings (e.g., Bahr et al., 2019; Cullinan & Biedzio, 2021). That said, very little qualitative work has been published in this area. The one exception is a line of inquiry showing that community college faculty and advisors doubt the usefulness of multiple measures and continue to feel an attachment to more traditional placement testing (Ngo et al., 2021).

In some cases, colleges or state education systems have gone a step further with placement reform, allowing students to make their own decisions about which classes to take. The model, called “informed self-placement,” involves college advisors providing guidance and information about different course options so that students can make informed enrollment decisions for themselves (Brathwaite et al., 2022). This guidance comes in the form of explanatory handouts, online resources, questionnaires and self-assessments, and individual and/or group advising sessions, to name a few (Toth, 2019). It is important to note that informed self-placement, or directed self-placement as many call it, “is not a single procedure, product, or algorithm, but rather a set of principles grounded in student choice that can be implemented in a variety of ways with varying consequences in local contexts” (Toth, 2019, p. 2). Even though informed self-placement presents a significant departure from traditional placement models, there is almost no scholarship about how it works and its impact on students (Kosiewicz & Ngo, 2020).

In 2013, the Florida College System (FCS) engaged in significant reform—involving both multiple measures and informed self-placement—to satisfy the requirements of Senate Bill (SB) 1720. As colleges prepared for rapid and extensive implementation, our research team became aware of large-scale impacts on the daily work experiences of administrators, faculty, and advisors. Motivated by these experiences of our FCS colleagues, as well as the aforementioned lack of scholarship in this area, we decided to take advantage of the unique opportunity before us to answer the following questions: (1) What does a newly implemented, informed self-placement system based on multiple measures look like in practice, according to the advisors who put it into place? and (2) What challenges and corresponding solutions emerged during implementation that other states and practitioners can learn from? In the research that follows, we answer these questions based on the perspectives of 275 advisors shared in the context of semi-structured focus group sessions.

## Literature Review

Although community colleges are open access institutions, they have historically relied upon standardized assessments to help place students in the appropriate level of coursework (Rutschow & Mayer, 2018). Students who are deemed “college ready” by such tests are placed directly into college-level courses, whereas those with demonstrated academic gaps are referred to remedial, or developmental education, coursework in subjects like mathematics and English.

While it is true that “all higher education involves sorting” (Hughes & Scott-Clayton, 2011, p. 329), scholars now question whether high stakes assessments are the best way to conduct that sorting. Taken together, the current body of literature clearly shows that relying solely on college placement tests to determine course enrollment decisions leads to frequent placement errors (Belfield & Crosta, 2012). In fact, as many as one in four community college students are assigned to the wrong level of math, and one in three students are assigned to the wrong level of English when standardized placement test scores are used alone (Scott-Clayton et al., 2014). Although placement tests can overestimate students’ academic preparation, underestimation is far more likely (Scott-Clayton, 2012). Interestingly, the FCS’s high stakes assessment tool—the Postsecondary Education Readiness Test [PERT]—is a better measure than some other standardized tests, but scholars Leeds and Mokher (2020) acknowledge that revising cut scores would improve the accurate placement of students.

Notably, the effects of placement testing are most acute for students of color. Klasik and Strayhorn’s (2018) analysis of college readiness measures among those at two- and four-year colleges confirms “racial differences in the predictive ability of college entrance exams” (p. 341), such that racial and ethnic minority students are considered less prepared by college placement tests than they actually are. Research done by Ngo and Melguizo (2021) specifically in the two-year sector similarly indicates that female students, Black students, and Latina/o/x students are especially vulnerable to being misplaced in developmental-level math courses due, in part, to placement testing policies.

The problem with erroneously placing students into developmental education is that the extra remedial coursework operates as an unnecessary roadblock. Developmental education courses are costly for students, both in terms of time and money, and there is a high likelihood that students will not make it through the lengthy course sequences, but instead drop out (Bailey et al., 2010, 2015; Complete College America, 2012). Indeed, a meta-analysis completed by Valentine and colleagues (2017) found overwhelming evidence that students placed into developmental education courses face significantly worse outcomes than their peers across three measures: passing college-level courses, earning college credit, and attaining a degree. Since the completion of that meta-analysis, additional research findings have only strengthened these conclusions. For instance, two recent studies indicate that the large number of students who are under-placed into developmental-level math end up discouraged and less likely to complete important degree-applicable credits and transfer-level STEM courses, ultimately impacting degree attainment at the associate’s and bachelor’s level (Ngo & Melguizo, 2021; Park et al., 2021).

### Multiple Measures

Considering the placement errors associated with standardized testing, reformers have begun to experiment with incorporating multiple measures of documented student achievement into the advising and placement process. Broadly speaking, multiple measures got their start in California in the late-1980s, partially in response to a court ruling on affirmative action (Burdman, 2012; Duffy et al., 2014). States like Wisconsin, North Carolina, New Jersey, Texas, and Connecticut were other early adopters (Burdman, 2012; Duffy et al., 2014; Kalamkarian et al., 2015). Today, more than half of colleges now rely on multiple measures to inform their advising processes (Rutschow & Mayer, 2018).

Advising systems based in multiple measures take on a variety of forms. Options range from simple waiver systems that exempt certain groups of students from placement testing to much more complex placement algorithms that calculate appropriate course recommendations according to the relative importance of various measures of past student achievement (Ganga & Mazzariello, 2019). Although the execution of multiple measures varies from state to state—and even college to college—two of the more common measures that are taken under consideration include student’s high school grade point average (GPA) and a consideration of the highest level of mathematics completed in high school.

According to analyses completed by Bahr and colleagues (2019), cumulative high school GPAs are the most reliable predictors of collegiate performance in math and English courses. Adding information about high school course completion (e.g., highest level of mathematics completed) refines placement practices even more. Scott-Clayton and colleagues (2014) attribute the utility of high school transcripts to the fact they reflect years of achievement and provide insight into such factors as student effort and motivation. That being said, placement tests still have some merit when used in concert with these other measures of demonstrated student success, particularly prior math achievement and high school GPA (Ngo & Kwon, 2015).

Numerous studies have been published, many within the past several years, on the efficacy of a multiple measures placement strategy. Research consistently finds that multiple measures improve placement decisions and long-term metrics of student achievement. For instance, work done by Cullinan and Biedzio (2021) demonstrates that students with low test scores, but strong high school GPAs and non-cognitive scores benefit from multiple measures placement that allows them to bypass developmental education. Indeed, those in this so-called “bump-up zone” are more likely to enroll in and complete college-level math and English courses within three years, compared to their peers whose course placements are dictated solely by test scores. Multiple measures seem also to reduce the achievement gap by increasing the number of female and racial and ethnic minority students who enroll directly in college-level courses (Hu & Hu, 2022; Park et al., 2018; Scott-Clayton et al., 2014).

### Informed Self-Placement

Informed self-placement is a related reform that first appeared in English and writing courses in the late 1990s (Royer & Gilles, 1998). While scholars and practitioners also refer to this practice as directed self-placement (e.g., Barnett & Reddy, 2017; Kosiewicz & Ngo, 2020; Toth, 2019) or guided self-placement (e.g., White & Newell, 2022), we chose to use “informed self-placement” here because we found Brathwaite and colleagues’, (2022) “guidance-choice continuum” to be an incredibly helpful way to understand this topic. Their approach plots the amount of choice and guidance evident in different placement methods along a spectrum, resulting in four quadrants: traditional placement (low choice, low guidance), self-placement (high choice, low guidance), informed self-placement (high choice, high guidance), and informed placement (low choice, high guidance).

In practice, informed self-placement uses available information to help students make their own advising decisions. Rather than relying solely on inflexible, standardized test cut points to assign students to a required course, students are shown how their varied high school experiences, grades, and test scores may lead to success or challenge in certain courses. In the end, it is up the students themselves to make the final decisions about their upcoming course schedule. That said, informed self-placement looks different from institution to institution due to the fact that colleges emphasize student engagement and guidance in the placement process to varying degrees (Morton, 2022).

Informed self-placement is much less pervasive than multiple measures placement, with Toth (2018) estimating in 2016 that only 1% of open access two-year colleges had officially experimented with offering their students this option. Perhaps because of this, research on informed self-placement, particularly in the context of two-year colleges, is limited. Nevertheless, informed self-placement appears to hold promise for improving student outcomes. Community colleges using this model have commonly noted a positive influence on course grades and completion rates of first-year writing courses (Klausman et al., 2016; Toth, 2018, 2019). Morton (2022) summarizes other benefits of informed self-placement, including: (1) the opportunity to foster student agency, (2) increased alignment with institutional priorities, (3) the fact that it represents a more holistic placement method, and (4) an improved learning environment due to student buy-in.

### Florida’s Advising Model

In light of all this emergent literature, the Florida legislature made the decision to majorly reform state colleges in 2013 via SB 1720. The hallmark of the bill was that it required all 28 FCS institutions to make placement tests and developmental education courses optional for two specific groups of “exempt” students – those who entered a Florida public school in 2003/04 or later and completed a standard high school diploma, as well as active-duty military personnel.

Developmental courses that remained on the schedule were redesigned for faster progression and made more applicable to students’ chosen fields of study. These redesigned courses were taught in new formats, or “modalities,” including modularized, compressed, contextualized, and/or co-requisite coursework. Colleges were not required to offer all four modalities simultaneously but were asked to provide options from which students could choose based on their schedule and preferred learning style.

Considering the increased number of choices facing students related to placement testing, developmental education, and new learning environments, revised advising procedures were also emphasized by the bill. Using the language of Brathwaite and colleagues’, (2022) “guidance-choice continuum,” SB 1720 expected colleges to increase both guidance and choice. Most significantly, advisors were asked to use multiple measures of documented student achievement when generating course recommendations. The following measures were provided as possible evidence of students’ academic preparation: work history, military experience, certifications, career interests, meta-major declaration, and additional assessment scores (2014 Fla. Statutes §1008.30, 2014). Importantly, these multiple measures were not used to dictate placement decision, but rather to help inform and guide exempt students in their decision making. Regardless of advisors’ recommendations, students could ultimately decide to enroll in the developmental or college-level math and English courses they deemed appropriate. While SB 1720 did not use the language of “informed self-placement,” that was indeed the outcome. In light of these substantial changes, reform in Florida provides a compelling case for studying new advising methods and the resulting challenges and successes.

### Theoretical Framework

In the literature, there are two prevailing approaches to advising: prescriptive advising and developmental advising. Prescriptive advising, much like a doctor-patient relationship, involves an advisor telling students exactly what courses they should take, with the understanding that the students will comply. Where prescriptive advising is transactional, developmental advising is reciprocal (Smith, 2002). In other words, developmental advising requires students to take initiative and share in the responsibility for their academic decisions. This kind of advising model is also distinctive in how it considers the whole student—including personal and educational factors—made possible by a deep advisor-advisee relationship (Crookston, 2009). While there are clear advantages to developmental advising, there are also barriers (e.g., large advising loads, lack of training and incentives, etc.) which have prevented it from becoming the dominant model (Barbuto et al., 2011).

The passage and implementation of SB 1720 represented a wholesale shift from prescriptive advising to developmental advising. Under the new informed self-placement model, students and advisors were intended to work together in order to get students enrolled in courses where they would be successful. As we move into a description of the study and the findings of our research, these benefits and challenges, as well as others, will be illuminated.

## The Study

This research is based on qualitative data gathered via 46 focus group sessions hosted by 19 different colleges between fall 2014 and spring 2019. Consistent with other embedded, single-case studies, we examined multiple units of analysis (275 individual advisors) embedded within the context of a larger case (the FCS) to explore advising practices during the implementation of SB 1720 (Yin, 2014).

### Data Collection

The FCS is comprised of 28 institutions spread across the state of Florida. Historically, these institutions identified as Florida’s community college system, but their course offerings have expanded in recent years to include some bachelor’s level degrees in fields like nursing, business, education, and information technology (Florida Department of Education, n.d.). To initiate this research project, we sent invitation emails to various administrators—including vice presidents, provosts, and deans—at all 28 FCS institutions in mid-2014. In some cases, we contacted known colleagues and in other cases, we relied on information made publicly available on the colleges’ websites to identify possible connections.

In the first year, ten of the 28 colleges agreed to participate. Over the next couple of years (fall 2015-fall 2018), additional invitations were sent to the colleges who had not yet responded. We also reached out to several institutions to schedule follow-up visits in years two through five, sometimes allowing us to talk with the same advisors again. A total of 19 different institutions provided us access to their advising departments.

After committing to host a site visit, participating colleges helped our research team recruit advisors for in-person, focus group sessions. Depending on availability and institutional size, we spoke with anywhere from two or three representatives to entire advising departments of 14 or 15 staff members during these campus visits, with an average participant count of six advisors per college. Advisors held various roles, with titles like “advising coordinator,” “director of advising,” “senior student success specialist,” “faculty counselor,” and “academic and career advisor.” Their self-reported work experience ranged from less than one year to more than 42 years, with five years of experience as the median response. Unfortunately, due to the large sample size (n = 275), we were unable link comments to specific advisors, and so comparisons by title, length of employment, and/or other demographic factors were not possible. Table 1 provides additional context regarding data collection between fall 2014 and spring 2019.

**Table 1:** Summary of Annual Data Collection Efforts

Year	Number of institutions	Number of Focus Groups	Number of advisors
2014–2015	10	13	84
2015–2016	8	9	47
2016–2017	6	6	35
2017–2018	9	9	51
2018–2019	9	9	58

To better understand how advising reform was implemented, we asked participants highly open-ended questions, such as: (1) How does advising happen at your institution?, (2) What model or philosophy of advising, if any, guides what you do in advising as a department?, (3) What information do advisors use to make course recommendations to students when standardized tests are unavailable?, and (4) Do you think students view their developmental education referral as accurate? In other words, do students seem to take your recommendations to heart? The audio from these focus groups was digitally recorded with permission from all participants and then transcribed into Word documents for later analysis.

### Data Analysis

Analysis occurred in multiple stages. The first round of analysis began in 2014 with a team of five researchers who used NVivo 12 to organize data according to an extensive, a priori coding framework. Initially, codes were descriptive and provisional (Saldaña, 2013), covering such themes as advising practices and the reactions of advisors to implementation. Over the next four academic years (i.e., fall 2015 to spring 2019) researchers coded the additional data collected on site visits to newly participating institutions, as well as those hosting follow-up visits, on a yearly basis. New codes were added, when necessary, to capture emergent themes related to sources of advising information, changing advising philosophies, and advising workload, among others. In an effort to engage in member checking, the research team drafted annual reports summarizing all main findings based on this coding work, which we sent to participating FCS institutions for participants to review.

A second cycle of coding was subsequently completed by the first author of this paper to interrogate the specific topics of informed self-placement and multiple measures. After searching the data for these key words and failing to find them, the author re-focused on instances where advisors spoke of choice and guidance. Some of the earlier codes, like sources of advising information and advising workload, were directly relevant to our research questions as is. Others codes had to be collapsed, reorganized, and redefined, which allowed subthemes like continued placement testing, group advising, and self-diagnostics to bubble up, providing the substance of our findings. In the section that follows, we present a small sampling of quotations that best represent the shared thoughts, opinions, and feelings of advisors spread across the FCS.

## Results

Due to the fact that SB 1720 did not use the language of “informed self-placement” or “directed self-placement,” we found that respondents did not use that language either. Nevertheless, advisors had plenty to say on the topic. For one, participants across all of the colleges strongly emphasized the ways in which developmental, informed self-placement was a departure from the prescriptive placement processes they facilitated prior to SB 1720. In the words of one advisor:

Advising appointments used to be very prescriptive… First semester, you know, especially if the student was all prep [i.e., developmental education], there was no discussion about major, there was no discussion about the career goals… It was, ‘Here’s your prep. This is where you start.’ [Now] the opportunity for that conversation exists, that opportunity to get the student to think more about their educational process.

Another advisor aptly described the new advising processes as “a collaborative effort between the student and the person who's advising them.” They continued, “it's a question of what do you want to do? … You kind of have to have a personal conversation with them to determine what it is that they want and what will fit in the lifestyle that they need.”

Consistent with the informed self-placement model, a third advisor added that these conversations give advisors the chance to “make recommendations related to enrollment.” That said, students are “driving the bus so, if they choose not to take it, that's up to them.” In other words, using the terminology of the “guidance-choice continuum” (Brathwaite et al., 2022), SB 1720 moved colleges away from a model of advising that was characterized by low information and low choice (i.e., “traditional placement”) towards a model characterized by high information and high choice (i.e., “informed self-placement”).

### Multiple Measures of Documented Student Achievement

These new conversations were organized around multiple measures of documented student achievement. As previously noted, SB 1720 required FCS advisors to consider the following information when generating placement recommendations: grade point averages, work history, military experience, participation in juried competitions, career interests, and degree major declaration (2014 Fla. Statutes §1008.30, 2014). Despite “multiple measures” being the term used widely in scholarly literature to describe this practice, only one advisor used the exact phrase in the entirety of the data we collected. Nevertheless, numerous advisors across the FCS described the inclusion of multiple measures in the advising and placement process using related language.

According to the data, high school performance, other standardized test scores (i.e., the ACT and SAT), and intended field of study were the most pervasive measures of documented student achievement used across the FCS, while juried competitions and discussions of military experience were largely absent. As an example of this, one advisor spoke about the significance of rigor, as it pertains to a student’s high school curriculum:

One of the conversations that we try to have is about the type of math they took in high school… Did you take an honors algebra course, or Algebra 2, or did you take math for college readiness? And what kind of grade did you get? Was it an A or a B or a C? … [With] this advising quick review sheet…, we're asking them to think about the kind of student they were in high school and what types of courses that they took.

#### Continued Placement Testing

Despite the many changes, a large number of participants indicated that placement test scores were still the most crucial component of the advising process, even under the new multiple measures model. As one advisor noted,

I try to get every single student to PERT test... I'll ask them high school grades. How did you do in English? If they're getting Cs in English, if they're getting Cs and Ds in math, I push very, very hard for a current assessment so that we can see a real read.

Advisors were more than willing to share their rationale for doing so, citing objectivity and ease. According to one advisor, “we push for the PERT as best as we can, because that gives you kind of an answer and kind of a benchmark, rather than just anecdotal information. You get a solid score.”

Generally speaking, some advisors saw value in the addition of multiple measures to advising, and some found them to be challenging to implement. Nevertheless, most agreed that multiple measures allowed them to take stock of the full range of student achievement, “rather than just putting them in something… that probably was a reflection of a bad day.”

#### Non-Cognitive Factors

Beyond the formal measures of achievement set forth by the Florida legislature, we also found that college advisors used non-cognitive factors to guide their recommendations. Advisors consistently acknowledged the importance of personal and professional commitments when helping students determine which courses were most appropriate for them. Illuminating this point, one advisor reflected:

I think you have to look at the student as a whole. You've got to see, are they ready? How many classes are they trying to take? What kind of classes did they take in high school? Do they have ACT and SAT scores? Are they working a full-time job? Do they have children? I think you need to look at all of that before you talk about classes.

These questions about work commitments and caretaking responsibilities were intended to help advisors determine how many competing priorities a student may be simultaneously juggling, and how much effort they might be able to put towards their coursework.

Advisors also noted the importance of dedication or motivation as another relevant factor that might shape student success in the classroom, regardless of past achievement. In the words of one advisor:

I see a lot of students that test low and they go into MAT 1033 [i.e., college-level math] and they make an A or a B. I think it's all about the student. You have to be dedicated to your studies. You know, they may struggle in math, but when they got that extra help, they got tutors, they went to the professor [they succeeded]. So, it's all about being disciplined in getting that help. So, I think it [placement testing] is good and it shows where they are at, at that time, but can they be successful? Of course. It's just all about dedication.

In the case of highly motivated students, advisors were grateful for the ability to guide them into courses they would not have been allowed to enroll in under the more inflexible, traditional placement model.

### Challenges of Informed Self-Placement

One of the most pressing challenges of informed self-placement articulated by participants was the time intensive nature of gathering and reviewing multiple measures of documented achievement, synthesizing the information, and then communicating the resulting recommendation to students. While advisors reported that advising sessions used to last approximately 15–20 min, appointments that occurred after the implementation of SB 1720 could take more than 60 min. As one advisor shared:

An appointment that normally took us in the past 20 minutes with a student, we can go anywhere from half an hour sometimes up to an hour, explaining the exemptions, talking about where they are with their levels… We have a little quiz that the reading in English and math departments have handed us… to show the students and say, ‘If you can't do these three problems in your head, you probably are not ready for intermediate algebra.’ Trying to give them a reality check, as to their capability in the areas. So, yeah, it has increased our advising time a lot.

Another challenge of multiple measures was that some advisors found themselves relying on self-reports of student achievement in the absence of high school information being readily available. In the words of an advisor, “Most of the time, it is self-reporting—‘How well did you do in high school?’—because we don’t always have access to their transcripts. So, we have to go by what they tell us when we’re trying to advise them.” Even when information was on file, some perceived the high school reports to be inflated or biased. To this point, one advisor pointed out that there are “differences in every high school and the standards that every high school brings.” Providing more details on the subject, another advisor elaborated:

I've had students… tell me that they made an A or a B in pre-calculus… They think that they are ready for college, and they're not… It goes back to the high school a lot, because those grades are not – they're not indicative of what they can [do]… They're inflated.

Ngo and colleagues (2021) reported similar distrust of high school data among college faculty, except in cases where it was supplemented by placement test scores.

Finally, some advisors found that providing guidance was difficult when many students arrived at their advising meeting with “pre-set ideas” after talking with friends, parents, and classmates. To this point, one advisor lamented that “it's getting harder and harder to get them to do anything other than what they already want to do.” Even when it seemed like students would take the advice of their advisor, they sometimes changed their minds after speaking with family and friends. As one advisor noted,

You would get them registered and you would feel good that, between the two of you, you've made the right decision. And ultimately, they would go home, discuss it with their family and, if you looked at their schedule a couple weeks later, they changed it… It was like banging your head against a brick wall.

Tracking student outcomes for those who disregarded their advisor’s professional opinions is beyond the scope of this study. However, it would be of great value for future research to document the successes or failures of those who enrolled in courses that college personnel did not initially recommend for them.

### Innovations

In the face of these challenges, college personnel found innovative ways to successfully implement informed self-placement based on multiple measures of student achievement. Our dive into the data uncovered several examples: group advising, advanced preparation, and advising guides. Each of these practices allowed advisors to better, and more quickly, serve the large number of students assigned to them.

#### Group Advising

One way that colleges managed the increased workload of informed self-placement was by offering group advising. This strategy allowed colleges to disseminate information to a large number of students without having to schedule every incoming student for an individual advising appointment during what is already “peak” season. In the candid words of an advisor, due to limited “manpower, we cannot see the current students and new students…So a lot of those things we try to narrow down so that we can share with everybody.” For most colleges that chose this method, group advising was carried out during orientation. As one advisor explained, “about half the orientation is spent in a group-advising session, broken out by meta-majors, and they talk about classes and get them registered for that first term.”

#### Advanced Preparation

While it is true that some colleges had advisors make course recommendations on the spot, many described a process whereby recommendations were generated in advance of an individual advising session or group orientation. In some cases, this preparation was done manually by advisors, and in other cases, it was computer automated. Either way, advanced preparation allowed advisors and/or computer software to make sense of multiple indicators of student achievement and generate thoughtful course recommendations outside of the time constraints of an advising appointment. As one advisor reported, “before they [students] come in, we have done all the research.” Elaborating on this point further, another described advanced preparation in the following way:

We get a list of the students that are going to be attending orientation, and then we look up their high school transcripts, their PERT scores, ACT scores, SAT scores, whatever … Before we even go to the orientation, we have them in certain classes based on the combination of different things.

While advanced preparation did not preclude advisors and students from making different choices later, particularly in an environment of informed self-placement, it did provide a starting point for conversation.

#### Self-Diagnostic Tools

Advisors did not share any clear solutions for the problem of students disregarding their professional opinions. We did, however, observe that advisors attempted to explain or justify their course recommendations to students through informal, self-diagnostic tools like study guides and sample questions from college-level classes. As one advisor explained, “I really love that little tool that our department has put together, just five math problems.” The five math problems were intended help students discern whether or not they feel comfortable with the material and are ready to jump straight into college-level mathematics courses.

These tools interplay with multiple measures of achievement, in that advisors can help students acknowledge their areas of weakness and make a plan to move forward. In the words of another advisor:

We can see their high school grades where maybe they just barely got by with Cs in math and/or English. I would say, ‘Based on your scores, what we're seeing is that your best option may be developmental education to get the foundation to be successful. But you have the right to opt out, and we have some examples that the departments gave us of what would be required in those courses.’ So usually, [I] pull those up to say ‘This is what will be required in this class.’

Advisors working for at least nine of the 19 participating colleges explicitly reported using these self-diagnostic tools to communicate the necessary prerequisites for success in college-level classrooms.

Again, the efficacy of this strategy is beyond the scope of the current research project. It is worth noting that a couple of advisors cast doubt as to whether these tools were successful in changing students’ minds. Anecdotally, one advisor shared that “We had that math test… I had a student look at it and he said, ‘It looks like Greek to me, but I don’t care, I still want to go into that class.’” Regardless of how compelling self-diagnostic tools are, at the very least they provide additional information for students to weigh while engaging in the informed self-placement process.

## Discussion

Following the passage of SB 1720, FCS institutions universally adopted informed self-placement based on multiple measures of documented student achievement. Implementation varied across the institutions, but commonly involved advisors talking with students about their high school experiences, test scores, and personal circumstances to determine an appropriate course schedule. As advisors did not refer specifically to informed self-placement by name, we unfortunately cannot speak to how much they understood about this placement method beyond the mandates of SB 1720. Nonetheless, their daily work was full of presenting numerous choices to students, alongside corresponding guidance. Interestingly, we did encounter evidence that advisors continued to push for students to complete the PERT, even when those tests were no longer required. This practice is not inherently problematic, so long as advisors do not ignore other measures of student achievement when making recommendations.

We also observed several challenges to the implementation of informed self-placement via multiple measures and solutions that emerged throughout the FCS. According to the advisors we spoke with, informed self-placement was time intensive and sometimes based on self-reports rather than official documentation of student achievement. In an unrelated study of advising within the FCS during the time of the COVID-19 pandemic, Mokher and colleagues (2024) similarly found that alternative placement methods were “time consuming” (p. 155) and that sometimes “the information that was needed to determine alternative placement wasn’t readily available” (p. 158). In our study, advisors also noted that recommendations were at risk of being undermined by the competing voices of family and friends.

Despite these challenges, SB 1720 has resulted in several positive outcomes. Not only did the changes increase the number of students attempting and passing college-level courses (Hu et al., 2019), they also generated cost savings for students and colleges alike (Mokher et al., 2021). These improvements are despite the fact that not all of the challenges of informed self-placement had corresponding solutions, and not all of the challenges were fully resolved. The increased workload, for example, was still quite difficult for many advisors to manage even with group advising. While these challenges clearly did not keep SB 1720 from improving student outcomes, they are worth keeping in mind as other states embark upon their own reforms.

Considering that the implementation of multiple measures can be time and labor intensive, our findings point to the importance of: (1) having sufficient advising staff to successfully facilitate multiple measures placement, (2) robust IT infrastructure to automate some of the more routine aspects of advising work, and (3) early and regular communication of the success of multiple measures placement to inspire confidence among those tasked with implementing it. Our findings also point to the potential value of training students as peer advisors. If students prioritize the voices of other students in the course selection process, sometimes over those of campus personnel, then perhaps training up a team of well-informed student leaders would be a worthy investment. This might allow accurate information to flow more easily from the institution to incoming students.

Furthermore, implementers of advising reform must be thoughtful in considering how changes to policies and procedures will impact students differentially. Research shows that informed self-placement and multiple measures placement both hold promise for improving student outcomes and increasing equity (Barnett et al., 2018; Cullinan et al., 2019; Kosiewicz & Ngo, 2020; Ngo et al., 2018; Toth, 2018). Unfortunately, not all students currently reap equal benefits of informed self-placement. Research suggests that female students and students of color tend to enroll in less rigorous classes than their white and male counterparts when choice is increased, perhaps because they underestimate their own academic abilities (Kosiewicz & Ngo, 2020; Park et al., 2018). Purnell and Burdman (2021) similarly find that first generation students and those with lower math confidence struggle to make optimal self-placement decisions. Those seeking to reform traditional advising structures must find ways to address this limitation in future designs.

## Conclusions and Directions for Future Research

Considering how understudied this topic is, there exist ample opportunities to increase scholarly understanding of informed self-placement. Several areas of potential growth are already documented in the findings section of this paper. In addition, we hope soon to explore the extent to which exempt students across various institutions in Florida understood that they had choice in the course selection process. In an extensive review of informed self-placement literature, Morton (2022) finds evidence of four types of informed self-placement: full student choice, limited choice, veiled choice, and informed self-placement without choice. It is likely that not all 28 FCS institutions provided students the same amount of autonomy in the course selection process, especially in light of research done by Brower et. al (2017) suggesting that Florida colleges varied in the extent to which they followed the letter and spirit of SB 1720, with some colleges exhibiting oppositional or circumventing behavior.

In addition, we suspect that advisors experienced changing perceptions or understandings of multiple measures during the five years of our study. In fact, other reports about the implementation of SB 1720 indicate that faculty, advisors, and administrators were generally skeptical or concerned about the negative repercussions of SB 1720 but eventually came to see the benefits for student success in time (Hu et al., 2016, 2017). While the purpose of this work was not documenting the phases of implementation or the process of change management, future work of this kind would be insightful and widely applicable to other educational reforms.

Finally, future research in this area might also consider the value that guidance adds to informed self-placement. Findings presented here cause us to question how much and under what circumstances guidance actually matters, particularly if students choose to disregard it. Relatedly, we wonder what constitutes good guidance and how different groups of students take that guidance under advisement.

## Data Availability

The data are not currently available.
